# Safety and efficacy of acupuncture for mild cognitive impairment: a study protocol for clinical study

**DOI:** 10.3389/fneur.2024.1346858

**Published:** 2024-03-15

**Authors:** Jae-Hong Kim, Jeong-Cheol Shin, Ae-Ran Kim, Bok-Nam Seo, Gwang-Cheon Park, Byoung-Kab Kang, Jeong-Soon Lee

**Affiliations:** ^1^Department of Acupuncture and Moxibustion Medicine, College of Korean Medicine, Dongshin University, Naju, Republic of Korea; ^2^Clinical Research Center, Dongshin University Gwangju Korean Medicine Hospital, Gwangju, Republic of Korea; ^3^Clinical Research Coordinating Team, Korea Institute of Oriental Medicine, Daejeon, Republic of Korea; ^4^KM Science Research Division, Korea Institute of Oriental Medicine, Daejeon, Republic of Korea; ^5^Department of Nursing, Christian College of Nursing, Gwangju, Republic of Korea

**Keywords:** acupuncture, computerized cognitive rehabilitation, RehaCom, mild cognitive impairment, study protocol

## Abstract

**Background:**

Mild cognitive impairment (MCI) is an intermediary condition between typical cognitive decline that occurs owing to aging and dementia. It is necessary to implement an intervention to slow the progression from MCI to Alzheimer’s disease. This manuscript reports the protocol for a clinical trial on the effect of acupuncture in patients with MCI.

**Methods:**

The trial will be a randomized, prospective, parallel-arm, active-controlled trial. Sixty-four patients with MCI will be randomized to the Rehacom or acupuncture group (*n* = 32 each). The participants in the acupuncture group will receive electroacupuncture at GV24 (Shenting) and GV20 (Baihui) and acupuncture at EX-HN1 (Sishencong) once (30 min) a day, twice per week for 12 weeks. The patients in the Rehacom group will receive computerized cognitive rehabilitation using RehaCom software once (30 min) daily, twice weekly for 12 weeks. The primary outcome measure is the change in the Montreal Cognitive Assessment Scale score. The secondary outcome measures are the Geriatric Depression Scale, Alzheimer’s Disease Assessment Scale-Korean version-cognitive subscale-3 scores, and European Quality of Life Five Dimensions Five Level Scale. The safety outcomes will include the incidence of adverse events, blood pressure, blood chemistry parameters, and pulse rate. The efficacy outcome will be assessed at baseline and at six weeks, 13 weeks, and 24 weeks after baseline.

**Discussion:**

The findings of this protocol will provide information regarding the effects of acupuncture on MCI.

**Clinical trial registration:**

https://cris.nih.go.kr/cris/search/detailSearch.do?search_lang=E&focus=reset_12&search_page=M&pageSize=10&page=undefined&seq=25579&status=5&seq_group=25579, KCT0008861.

## 1 Introduction

Mild cognitive impairment (MCI), an intermediary condition between typical cognitive decline owing to aging and dementia, is a slight impairment in memory or other cognitive domains. MCI has no significant effects on daily living and does not meet the diagnostic criteria for Alzheimer’s disease (AD) (1, 2). In China, the prevalence of MCI increases with age from 11.9% in the 60s to 33.1% in the 90s or older (3). MCI in older adults is at a higher risk of progressing to AD (4). A previous five-year follow-up study reported that the annual conversion rates to dementia among older Chinese adults were 6.3 and 1.6% at baseline for participants with MCI and normal cognitive ability, respectively (5). The cognitive ability of patients with MCI may stabilize or return to normal status; however, progression to AD is more common. The annual incidence rates of MCI and dementia were reported to be 4.1 and 5.17%, respectively, in a previous cohort study. Notably, the rate of conversion to probable AD is high in the United States (241.3/1000 person-years), and MCI reverts to normal cognition in only few cases (6). AD is an irreversible condition; thus, early detection of MCI and the prevention of its progression may be urgent and important in the management of AD (7).

The demand for effective interventions for treating MCI is growing; however, no drugs supported by high-quality evidence have been recommended for treating MCI (8). Nevertheless, new management measures, such as regular exercise, cognitive training, and the management of modifiable risk factors, may aid in effectively managing MCI (9, 10).

Acupuncture, a technique involving the application of manual and electrical stimulation after inserting needles into acupoints, has been used for treating cognitive decline, including MCI (11, 12). The therapeutic effects of acupuncture on MCI have been reported in several systematic reviews and meta-analyses. Acupuncture was safe and effective for MCI when used as an alternative or adjunctive treatment compared with that observed when sham acupuncture, medication, and usual care were used as treatment (12–16). A systematic review of neuroimaging studies revealed that acupuncture may have an effect on the regulation of the central executive network, salience network, and default mode network, especially in the prefrontal cortex, cingulate cortex, and hippocampus in patients with MCI (17). Moreover, acupuncture was found to have modulatory effects on various regions of the brain in patients with MCI in a regional homogeneity-based meta-analysis (18). The potential therapeutic mechanisms of acupuncture include the restoration of the blood–brain barrier, downregulation of Aβ accumulation and tau protein phosphorylation, enhancement of synaptic plasticity, reduction of neuroinflammation, improvement of mitochondrial activity, and reduction of neuronal apoptosis (19). In a network meta-analysis that compared the efficacy of nine interventions for MCI, acupuncture and music therapy were found to have improved cognitive function in patients with MCI; thus, these treatment options may be favored for treating MCI (20).

Several studies have been conducted on the efficacy of acupuncture for MCI; however, the methodological quality of these studies has been insufficient in providing convincing evidence (12–18). Our manuscript describes the protocol for a rigorously designed clinical study that aims to explore the effects of acupuncture on MCI. The findings of this trial will provide evidence regarding the efficacy of acupuncture for MCI.

## 2 Methods and analysis

### 2.1 Objective

1. We intend to investigate the efficacy of acupuncture for MCI by comparing its effect on cognitive improvement with that of computerized cognitive rehabilitation (CCR).
2. We intend to evaluate the ability of acupuncture to improve the quality of life, memory, and depressive symptoms in patients with MCI.
3. We also intend to explore the safety of acupuncture in patients with MCI.

### 2.2 Hypothesis

1. The acupuncture group compared with the control group will show a better effect in improving cognitive ability.
2. Significant improvement in quality of life, memory, and depressive symptoms will be observed in the acupuncture group.
3. EA is a safe therapy for patients with MCI.

### 2.3 Study design and setting

**Figure 1:**
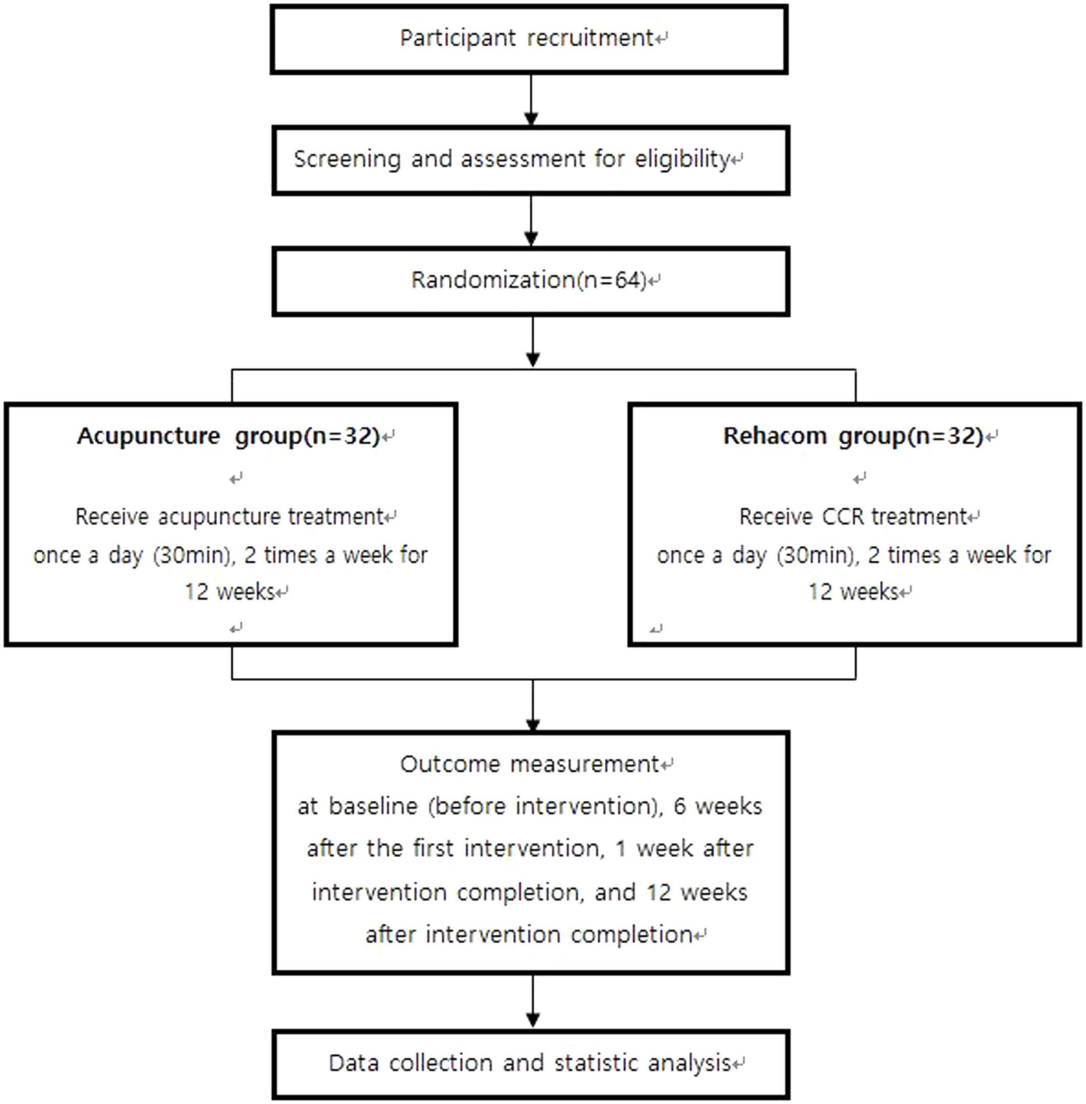
Flow chart of this study.

The trial will be a randomized, single-center, prospective, parallel-arm, active-controlled trial. Sixty-four patients with MCI will be randomized to the Rehacom or acupuncture group (*n* = 32 each). All patients will be educated on self-management and exercises techniques. The acupuncture group will receive acupuncture treatment once daily, twice weekly for 12 weeks, whereas the Rehacom group will receive CCR treatment using RehaCom software once a day, twice per week for 12 weeks. The duration of each session of acupuncture treatment and CCR treatment will be 30 min. The efficacy outcome will be assessed at baseline (week 0), 6 weeks after baseline (week 7), 13 weeks after baseline (week 13), and 12 weeks after baseline (week 24). Table 1 presents the schedule for assessment, enrollment, and intervention. Figure 1 presents the flowchart of this study. Our manuscript complies with the Standard Protocol Items: Recommendations for Interventional Trials Reporting Checklist (Additional file 1) (21).

**Table 1:** Trial schedule according to the SPIRIT guidelines.

	Study period						
	Enrolment	Allocation	Post-allocation				Close-out
Timepoint	Screening		Visit1-12	Visit13	Visit14-24	Visit25	Visit26
	Week −2 to 0		1–6	7	7–12	13	24
Enrolment							
Informed consent	X						
Sociodemographic profile	X						
Medical history	X						
Vital signs	X	X	X	X	X	X	X
Inclusion/exclusion criteria	X						
Allocation		X					
K-MMSE, Global Deterioration scale	X						
Interventions							
Acupuncture or computer-based cognitive rehabilitation			X	X	X	X	
Education on exercise and self-management			X	X	X	X	
Assessments							
Change of medical history			X	X	X	X	X
Occurrence of adverse events			X	X	X	X	X
Blood chemistry test	X					X	
MoCA-K	X	X		X		X	X
GDS	X	X		X		X	X
ADAS-K-cog 3		X		X		X	X
EQ-5D-5L		X		X		X	X

SPIRIT, Standard Protocol Items Recommendations for Interventional Trials; K-MMSE, Korean Mini-Mental State Examination; MoCA-K, Korean version of the Montreal Cognitive Assessment scale; GDS, Geriatric Depression Scale; ADAS-K-cog 3, Alzheimer’s Disease Assessment Scale-Korean version-cognitive subscale-3; EQ-5D-5L, European Quality of Life Five Dimension Five Level scale.

### 2.4 Participant recruitment

Recruitment will be conducted at the Dongshin University Gwangju Korean Medicine Hospital, Republic of Korea, using local newspaper advertisements, community posters, and advertisements on the Internet. Potentially eligible individuals will receive an explanation regarding the aims and objectives of the trial from the investigator and clinical research coordinator (CRC). Only the participants who voluntarily sign the informed consent form before participation will be included in the trial. Patients will be screened using the inclusion and exclusion criteria. The CRC will explain the schedule of the next visit at each visit and adjust the schedules to ensure compliance with the protocols.

### 2.5 Inclusion

(1) Elderly adults aged between 55–85 years, (2) patients with memory problems persisting for at least three months who meet the diagnostic criteria for MCI (22), (3) patients with a Global Deterioration Scale score of 2 or 3, (4) patients with a Korean Mini-Mental State Examination score of 20–23, (5) patients with a Korean version of Montreal Cognitive Assessment scale (MoCA-K) score of 0–22, (6) patients with a Geriatric Depression Scale (GDS) score of 0–18, (7) patients with at least six years of education or adequate proficiency in Korean language to facilitate reliable study assessments, and (8) patients who are capable of providing informed consent voluntarily.

### 2.6 Exclusion criteria

(1) Diagnosis of vascular dementia in accordance with the NINDS-AIREN criteria or diagnosis of AD according to the NINCDS-ADRDA criteria; (2) a history of structural brain diseases that could result in cognitive disorder, such as congenital mental retardation, intracranial space-occupying lesions, traumatic brain injury, or stroke; (3) a history of serious illness, such as Huntington disease, Parkinson’s disease, cancer, central nervous system, liver, cardiovascular, and kidney diseases, and multiple sclerosis; (4) a history of neurological disease within the preceding 12 months confirmed using brain magnetic resonance imaging or computed tomography; (5) a history of alcohol, drug abuse, or mental illness (depression, serious anxiety, or schizophrenia) within six months prior to screening; (6) currently receiving treatment for MCI, such as Korean medicine treatment, drug, or cognitive training within 4 weeks prior to screening; (7) difficulties with outcome measures due to visual and hearing impairments; (8) unfit to receive EA owing to the presence of blood-clotting abnormalities, such as scalp infection, the presence of a pacemaker, or hemophilia; (9) pregnancy or breastfeeding; (10) participation in another study within 8 weeks prior to screening or current participation in another study.

### 2.7 Dropout and violation criteria

Dropout criteria: (1) incidence of any serious adverse event (SAE), (2) incomplete data that could impact the results, (3) withdrawal of consent for participation, or (4) exclusion from the study deemed necessary by the Institutional Review Board (IRB) or principal investigator (PI).

Exclusion from the per-protocol set analysis will be mandated for participants who demonstrate (1) remarkable protocol violation or serious deviation in protocol implementation and (2) an intervention compliance rate of <75% (completed less than 18 of the 24 scheduled intervention sessions).

### 2.8 Randomization

The participants will be randomized to the Rehacom or acupuncture group (*n* = 32 each) after a screening visit in accordance with random numbers generated using SPSS (version 21.0; IBM). The random code will be sealed in an opaque envelope and stored in a cabinet with a double lock.

### 2.9 Implementation

Allocation and random number generation will be conducted by an independent investigator who is not involved in this study. The enrollment procedures will be performed by the CRC.

### 2.10 Blinding

Statisticians, assessors, and data analysts will be blinded to the allocation during the trial process. Unblinding will be permitted with the approval of the IRB if SAEs occur. Only investigators with no conflicts of interest will be involved in the trial to avoid bias. All researchers will be trained in blinding procedures prior to participation.

### 2.11 Interventions

#### 2.11.1 Acupuncture treatment

Acupuncture treatment will be provided by licensed Korean medical doctors. The physicians will use the same technique to ensure compliance with this protocol. We adopted the acupuncture treatment methods, including the stimulation parameters of EA, based on those used in our preliminary study and acupuncture clinical studies for MCI (13, 16, 23, 24). The participants in the acupuncture group will receive electroacupuncture (EA) at GV24 (Shenting) and GV20 (Baihui), along with acupuncture at EX-HN1 (Sishencong). Single-use sterile acupuncture needles (DB106; size, 0.25 × 30 mm; Dongbang Medical Co., Ltd., Seongnam, Republic of Korea) and an EA stimulator (STN-330; Stratek, Co., Ltd., Anyang, Republic of Korea) will be used in the trial. In the supine position, the anterior EX-HN1, GV20 (Baihui), and GV24 (Shenting) will be punctured in the anterior direction. The right, posterior, and left EX-HN1 (Sishencong) will be punctured in the direction of GV20 (Baihui). After the acupuncture needles are inserted, they will be retained in the acupoints for 30 min without manual stimulation. EA will be applied at GV24 (Shenting) and GV20 (Baihui) using the following parameters: voltage, 0–5 V; frequency, 3 Hz; and continuous waves. Participants in the acupuncture group will receive 24 sessions of acupuncture twice weekly for 12 weeks, with each session lasting for 30 min.

### 2.12 Computerized cognitive rehabilitation treatment

Patients in the Rehacom group will receive CCR treatment in the seated position using RehaCom software. Rehacom consists of a large screen and an input panel that is used to train elderly individuals in several cognitive domains, including attention, verbal memory, delayed and episodic memory, and processing speed, as well as executive functions. All participants will receive training on the same tasks for equal durations (min/task) in each session. The training will be initiated at the beginner level of the training task, and the difficulty levels will be adjusted to meet the participant’s ability (23, 25). Participants in the CCR group will receive 24 sessions of CCR twice weekly for 12 weeks, with each session lasting for 30 min.

Management of modifiable risk factors, such as smoking, diabetes, hypertension dyslipidemia, cerebrovascular and heart disease, lower education level, regular exercise, rural residential status, and independent living, can be effective in the improvement of cognitive function in patients with MCI (3, 10). Therefore, all patients will be educated on self-management and exercise techniques. The CRC will monitor the participants’ medical condition at each visit, and the visit schedule will be changed in accordance with the participants’ request or the judgement of the PI to increase compliance with the study protocol. All participants will continue using currently received medications and treatments that do not affect their cognitive function during the trial. However, the participants will not be permitted to receive other treatments to improve the symptoms of MCI.

### 2.13 Outcome measurements

#### 2.13.1 Efficacy outcome

The primary outcome measure is the between-group difference in changes in MoCA-K scores 13 weeks after baseline. The secondary outcome measures of this study are (1) the between-group differences in changes in the MoCA-K scores 6 weeks after baseline; (2) the between-group differences in changes in the MoCA-K scores 24 weeks after baseline, and (3) the changes in the GDS, Alzheimer’s Disease Assessment Scale-cognitive subscale-3 (ADAS-K-cog 3), and European Quality of Life Five Dimension Five Level scale (EQ-5D-5L) scores 6, 13, and 24 weeks from baseline.

MoCA, a screening instrument that is widely used to detect MCI, is used to measure the changes in cognitive function. It is a 30-point test that can evaluate the cognitive domains of orientation to place and time, visuospatial/executive function, short-term memory recall, concentration, attention, working memory, and language (26, 27).

ADAS-K-cog 3 is used to evaluate memory. It includes three tasks of the ADAS-K-cog 11 (orientation, word recall, and word recognition), which demonstrate no ceiling effects in MCI (28).

GDS is used to identify depression as it can distinguish between the symptoms of depression and dementia in elderly people. This is a widely used screening instrument for evaluating depressive symptoms in elderly individuals (29).

EQ-5D-5L is a commonly used health utility instrument to explore quality of life (30). The quality weight of EQ-5D-5L was based on a previous study (31).

#### 2.13.2 Safety outcome

Safety assessments will be conducted by comparing the occurrence of adverse events (AEs) and the changes in pulse rate, blood pressure, and blood chemistry parameters between the two groups.

### 2.14 Adverse events

The possible AEs include bleeding, skin irritation, dizziness, pallor, local hematoma, and fainting. The CRC will record AEs occurring during the trial period, including the severity, date and time of occurrence, treatment process, and relationship between the AE and intervention. All AEs and SAEs will be reported to the PI and IRB and monitored until they stabilize. The participants will be compensated according to the applicable regulations in the event of SAEs and AEs related to our treatment.

### 2.15 Quality control

Experts in acupuncture, MCI, statistics, rehabilitation, and methodology developed and reviewed this protocol. All researchers will receive intensive training before commencing the trial to fully understand the protocol and standard operating procedures (SOPs). An independent clinical research associate will evaluate all documents related to this trial and monitor the trial to ensure that this study is conducted according to SOPs and protocols. Any revision of the protocol will be approved by the IRB.

### 2.16 Sample size estimation

We adopted sample sizes from a previous study that was similar to our study design and aimed to investigate the efficacy of acupuncture for MCI using the MoCA score as the primary outcome. In that previous study (32), the sample size was calculated assuming an expected curative effect of 90% in the acupuncture group and 67.9% in the control group, a two-sided alpha level of 0.05, and a statistical power of 0.8.


$$n=\frac{8pq}{\left(p1-p2\right)2}$$

In the equation, p1 and p2 represent the original curative effect (the rate of cognitive training) and expected curative effect (the rate of acupuncture treatment) of 67.9 and 90%, respectively. p = $\frac{p1+p2}{2}$ = 0.79, q = $\frac{1-p1+1-p2}{2}$ = 0.21.

The sample size was estimated as 27 participants per group, and assuming a maximum dropout rate of 15%, 64 participants (32 in each group) will be included in the trial.

### 2.17 Statistical analysis

An independent statistician will analyze the final data using SPSS software (version 21.0, IBM). A full analysis set will be conducted to assess the efficacy of the treatment, and missing data will be inputted using the last observation carried forward method. The significance level will be set at 5% (two-tailed). Categorical variables will be expressed as percentages or frequencies, whereas continuous variables will be expressed as mean and standard deviation or median with an interquartile range. Interim analysis will not be performed.

Friedman test or a one-way repeated-measures analysis of variance for intragroup comparisons and analysis of covariance, as well as an independent *t*-test or Mann–Whitney U test will be used for intergroup comparisons to analyze the changes in efficacy outcomes. Sub-analyses will be conducted according to age (under 70 and over 70 years old).

The occurrence of AEs between the two groups will be compared using the chi-square test or Fisher’s exact test. The Mann–Whitney U test or independent *t*-test will be used for intergroup comparisons to analyze the changes in blood chemistry parameters, blood pressure, and pulse rate.

### 2.18 Data management and confidentiality

All documents will be anonymized to conceal the names of participants, and identification codes will be assigned. An investigator who is blinded to the group assignment will record all data in an Excel file. These electronic data will be password-protected. All identification records and data can be accessed after receiving approval from the IRB. Raw data will be kept for three years after the study. Participants will voluntarily provide written informed consent for the dissemination of their individual details.

## 3 Discussion

Some supporting evidence has been provided for the use of acupuncture for MCI; however, the effects of acupuncture on the cognitive ability of patients with MCI are controversial owing to low methodological quality. We designed a rigorous clinical trial to provide high-quality evidence for the effects of acupuncture on MCI. The design of this trial is based on previous studies (12–16, 23) and our preliminary study (24).

A preliminary clinical study was conducted to compare the effects of CCR combined with EA and CCR alone to explore the effects of EA on MCI. EA combined with CCR, which was applied simultaneously with EA at Baihui (GV20), Sishencong (EX-HN1), Fengchi (GB20), and Shenting (GV24) and Rehacom software therapy for 30 min in a sitting position, had no positive add-on effects on the depressive symptoms, quality of life, activity of daily life, and cognitive ability in patients with MCI in the preliminary study (24). This trial has some improvements compared with the preliminary study. First, a larger sample size will be used to explore the effects of acupuncture on MCI. The preliminary study was a pilot study (*n* = 18 in each group), whereas this trial will have a sample size (of 32 in each group) based on that of a previous study. Second, the acupuncture treatment method has been modified based on the results of the preliminary study. Acupoint specificity, psychological factors, and duration of treatment can affect the efficacy of acupuncture. The choice of acupoints plays a crucial role in ensuring the positive therapeutic effects of acupuncture. GV24 (Shenting), EX-HN1 (Sishencong), and GV20 (Baihui), the most commonly used acupoints in previous studies, will also be used in this trial (16, 20). These acupoints are connected to the brain and play important roles in cognition (20). The participants in the preliminary study received EA stimulation at GV24, GV20, EX-HN1, and GB20 in the seated position. However, the participants showed resistance to EA treatment owing to discomfort in the seated position and the large number of EA stimulations. Therefore, in this study, EA will be performed at GV20 (Baihui) and GV24 (Shenting) (23) and acupuncture will be performed at EX-HN1 (Sishencong) in the supine position. Moreover, body acupuncture will not be adopted. MCI is a neurodegenerative disease that progresses slowly; therefore, the treatment duration was changed from 8 weeks to 12 weeks to achieve sufficient therapeutic effects based on the findings of previous studies (21, 32–34). Third, the interventions of the test and control groups were changed from EA combined with CCR and CCR to EA and CCR, respectively, to explore the effects of EA on MCI rather than add-on effects. Previous studies exploring the efficacy of acupuncture for MCI used sham acupuncture, pharmacological intervention, and usual care as interventions in the control group (12, 13, 15, 32–34). Sham acupuncture has been used in clinical studies on acupuncture to exclude the placebo effects. However, recent studies have shown that sham acupuncture should not be considered the same as placebo owing to the unexpected therapeutic effects of sham acupuncture at the same location (35, 36). There are no recommended drugs for MCI with a high level of evidence (9, 10). Therefore, CCR using Rehacom software will be used as the treatment in the control group. CCR may have positive effects on memory, attention, and executive function in patients with MCI (37, 38). As Rehacom provides standard tasks with immediate feedback, it is useful for the treatment of MCI and clinical trials (23). Fourth, the primary efficacy outcome was changed from ADAS-K-cog to the MoCA-K. MoCA has been used as the primary outcome in recent studies exploring the effects of acupuncture on MCI (16, 32, 34, 39). In addition, MoCA has also been used to evaluate early cognitive decline and cognitive reserve (CR). CR, a concept based on brain plasticity, counteracts the effects of brain damage and aging (40). CR has been proposed as a potential protective factor against dementia or MCI (41).

Our trial has some limitations. First, all participants will receive the same acupuncture treatment without syndrome differentiation as this study will follow a fixed acupuncture regimen. Syndrome differentiation plays a key role in the clinical decision-making process in traditional Chinese medicine and Korean medicine. Personalized herbal medicine and acupuncture treatment are prescribed to each patient based on the syndrome differentiation (42). Thus, the fixed acupuncture regimen may not demonstrate the efficacy of acupuncture completely. Second, only an active control group using CCR will be used in this study instead of a negative control group using usual care or sham acupuncture owing to limitations in research funding and research period. Third, neuroimaging outcomes will not be adopted to explore the therapeutic mechanism of acupuncture for MCI owing to the circumstances of our research institution.

Nevertheless, the findings of this protocol will provide evidence regarding the safety and efficacy of acupuncture for treating MCI and contribute to the development of an optimal standard acupuncture treatment method, thereby increasing the usefulness of acupuncture in the treatment of MCI.

## Ethics statement

This protocol (version. 1.0) has been approved by the IRB of the Dongshin University Gwangju Korean Medicine Hospital (date: August 22, 2023; Number: DSGOH-2023-004). This trial will adhere to the Korean Good Clinical Practice guidelines and the Declaration of Helsinki principles. The risks and purpose of the study will be explained to the participants. Only participants who provide written informed consent before participation will be included in this study. The final data will be reported to the IRB. The findings of this protocol will be published in a reputed journal following peer review.

## Author contributions

J-HK: Conceptualization, Funding acquisition, Investigation, Project administration, Supervision, Validation, Writing – original draft, Writing – review & editing. J-CS: Conceptualization, Investigation, Project administration, Supervision, Validation, Writing – original draft, Writing – review & editing. A-RK: Data curation, Methodology, Resources, Writing – review & editing. B-NS: Data curation, Methodology, Resources, Writing – review & editing. G-CP: Resources, Software, Visualization, Writing – review & editing. B-KK: Formal analysis, Writing – review & editing. J-SL: Formal analysis, Writing – review & editing.
