# Supplementary material for: Safety and efficacy of acupuncture for mild cognitive impairment: a study protocol for clinical study
Source: Front Neurol. 2024 Mar 15;15:1346858. doi: 10.3389/fneur.2024.1346858 (PMC10979737; doi:10.3389/fneur.2024.1346858)
Supplement: Supplementary file 1 [file Data_Sheet_1.PDF]

IRB approval

The Institutional Review Board of DongShin University Gwangju Korean Medicine Hospital approved this study (DSGOH-2023-004)

Institutional Review Board of DongShin University Gwangju Korean Medicine Hospital

Tel : 82-62-350-7115 FAX : 82-62-350-7551 141, Wolsan-ro, Nam-gu, Gwangju City, 61619, Republic of Korea

Certificate of Approval

<table>
<tr><td rowspan="2">Receiver</td><td>Principal Investigator</td><td>Name</td><td colspan="2">Jae-Hong Kim</td><td>affiliation</td><td colspan="2">College of Korean Medicine, Dong-Shin University</td></tr>
<tr><td colspan="2">Support Organization</td><td colspan="3">Ministry of Science and ICT</td><td>Position</td><td>professor</td></tr>
<tr><td>IRB No.</td><td colspan="7">DSGOH-2023-004</td></tr>
<tr><td rowspan="2">Title of clinical trial</td><td colspan="5" rowspan="2">A randomized, prospective single center clinical trial to compare the cognitive improvement effects of acupuncture and computerized cognitive rehabilitation for mild cognitive impairment</td><td colspan="2">Protocol No. DSS-SSH</td></tr>
<tr><td colspan="2">Ver. 1.0</td></tr>
<tr><td rowspan="4">Type of research</td><td colspan="7">□ Genetic testing □ Genetic research □ Gene therapy □ Gene bank
□ Use of personal data from medical records and biological materials
□ Survey □ Observational study □ Human tissue and blood research
■ Clinical trial (Drug, Medical device, biological medicine □ ETC ( )</td></tr>
<tr><td rowspan="3">Clinical trial</td><td>Product</td><td colspan="2">Acupuncture, electrical stimulator</td><td>Model</td><td colspan="2">DB106, STN330</td></tr>
<tr><td colspan="6">■ Academic use □ Obtain a domestic license (KFDA)
□ Obtain a foreign country license (Country:)</td></tr>
<tr><td>Phase</td><td colspan="5">□ Phase 1 □ Phase 2 □ Phase 3 □ Phase 4 ■PMS</td></tr>
<tr><td>Type of review</td><td colspan="7">■ Regular meeting □ Special meeting □ Emergency meeting</td></tr>
<tr><td>Date of review</td><td colspan="7">August 21, 2023</td></tr>
<tr><td rowspan="7">Approved document list</td><td colspan="3">■ Protocol (New)</td><td colspan="4">■ Principal investigator’s curriculum vitae</td></tr>
<tr><td colspan="3">□ Protocol(Supplementation)</td><td colspan="4">■ Informed consent</td></tr>
<tr><td colspan="3">□ Protocol(Revision)</td><td colspan="4">■ Case report form</td></tr>
<tr><td colspan="3">□ Interim report</td><td colspan="4">■ study advertisements</td></tr>
<tr><td colspan="3">□ Completion study report</td><td colspan="4">■ Compensation for Clinical Trial Participation'</td></tr>
<tr><td colspan="3">□ Clinical study report</td><td colspan="4">□ Documents provided to participants</td></tr>
<tr><td colspan="3">□ Continuous review</td><td colspan="4">■ ETC(Investigator’s brochure)</td></tr>
<tr><td>Result</td><td colspan="7">■ Approve □ Corrective approve □ Complementary approve □ Reject
□ Suspend for approved clinical trial</td></tr>
<tr><td>Date of approve</td><td colspan="3">August 22, 2023</td><td colspan="2">The date of validity</td><td colspan="2">February 20, 2024</td></tr>
<tr><td>Reporting period</td><td colspan="7">□ 3month □ 5month ■ 1year □ ETC( )</td></tr>
<tr><td>Verdict</td><td colspan="7">Report to the IRB immediately when the protocol changed by Food and Drug Administration regulations.</td></tr>
</table>

August 22, 2023

Chairman of the Institutional Review Board of DongShin University Gwangju Korean Medicine Hospital

This is to certify that the information contained herein is true and correct as reflected in the records of the DSUGOH Institutional Review Board (DSUGOH IRB).

We certify that DSUGOH IRB is in full compliance with Good Clinical Practice as defined under the Korea Food and Drug Administration(KFDA) regulations and the International Conference on Harmonisation (ICH) guidelines.

Institutional Review Board of DongShin University Gwangju Korean Medicine Hospital
Tel : 82-62-350-7114 FAX : 82-62-350-7551 141, Wolsan-ro, Nam-gu, Gwangju City, 61619, Republic of Korea

ALL DSUGOH IRB APPROVED INVESTIGATORS MUST COMPLY WITH THE FOLLOWING :

1. Conduct the research as required by the protocol.
2. Use only the Consent Form bearing the DSUGOH IRB " APPROVED" stamp.
3. Provide non-Korean speaking subjects with a certified translation of the approved Consent Form in the subject's first language. The translated version must be approved by the DSUGOH IRB.
4. Obtain pre-approval from the DSUGOH IRB of any changes in the research activity (except when necessary to protect human subjects; immediately report to the DSUGOH IRB any such emergency changes for the protection of human subjects).
5. Report to the DSUGOH IRB the death, hospitalization, or serious illness of any study subject.
6. Promptly report to the DSUGOH IRB any new information that may adversely affect the safety of the subjects or the conduct of the trial.
7. Provide reports to the DSUGOH IRB concerning the progress of the research, when requested.
8. Obtain pre-approval of study advertisements from the DSUGOH IRB before use.
9. Conduct the informed consent process without coercion or undue influence, and provide the potential subject sufficient opportunity to consider whether or not to participate.

This is to certify that the information contained herein is true and correct as reflected in the records of the DSUGOH Institutional Review Board.

We certify that DSUGOH IRB is in full compliance with Good Clinical Practice as defined under the Korea Food and Drug Administration(KFDA) regulations and the International Conference on Harmonisation (ICH) guidelines.
